# Fostering Self-Regulated Learning in Online Environments: Positive Effects of a Web-Based Training With Peer Feedback on Learning Behavior

**DOI:** 10.3389/fpsyg.2022.813381

**Published:** 2022-04-25

**Authors:** Henrik Bellhäuser, Patrick Liborius, Bernhard Schmitz

**Affiliations:** ^1^Department of Psychology, Faculty 02: Social Sciences, Media, and Sports, Johannes Gutenberg-University Mainz, Mainz, Germany; ^2^Institute of Entrepreneurship, University of Liechtenstein, Vaduz, Liechtenstein; ^3^Institute for Psychology, Department of Human Sciences, Technical University of Darmstadt, Darmstadt, Germany

**Keywords:** self-regulated learning, web-based training, peer feedback, training evaluation, learning diary

## Abstract

Although training in self-regulated learning (SRL) is effective in improving performance, human trainers can reach only a few people at a time. We developed a web-based training for potentially unlimited numbers of participants based on the process model of SRL by Schmitz and Wiese (2006). A prior study (Bellhäuser et al., 2016) observed positive effects on self-reported SRL and self-efficacy. In the present randomized controlled trial, we investigated an improved version of the web-based training, augmented by the application of peer feedback groups. Prospective university students in an online mathematics preparation course were assigned randomly to one of four experimental conditions: Group D (diary), group TD (training + diary), group TDP (training + diary + peer feedback group), and group C (control). Complete data was obtained for 136 participants (78.8% male; *M* = 19.8 years). The learning diary was intended to trigger goal setting, planning, and self-motivation in the morning and reflection in the evening. The web-based training consisted of three lessons (approximately 90 min each) with videos, presentations, self-tests, and exercises. In the peer feedback condition, participants were randomly assigned to groups of five persons each and used a bulletin board to discuss pre-defined topics related to the content of the web-based training. Outcome measures included a test of declarative SRL knowledge, an SRL questionnaire, a general self-efficacy scale, log file data, and a mathematics test. Results showed positive effects for the web-based training, particularly when combined with peer feedback on both SRL knowledge and SRL questionnaires, self-efficacy, and on objective time-investment, but not on the mathematics test. The learning diary did not exhibit positive effects. We conclude that additional peer-feedback seems to be a useful supplement to web-based trainings with comparably low organizational costs.

## Introduction

Self-regulated learning (SRL) has been shown to be highly relevant to academic achievement not only in secondary schools (Dignath and Büttner, 2008) but also in particular at university level (Richardson et al., 2012). University students need to work independently and decide every day what to learn, when and where to learn, and which learning strategies they want to apply. Due to their high workload, students need to plan their learning process based on their personal goals. Further, as setbacks and failures are common experiences, students also have to regulate their motivation. In particular, SRL strategies are a requirement for the success of students in computer-based learning environments (CBLE) (Broadbent and Poon, 2015). However, many students appear to have difficulties regulating their own learning process. Fortunately, researchers have demonstrated that training in SRL strategies is possible and that participants in SRL training substantially increase their academic performance (Theobald, 2021). Most approaches to fostering SRL apply face-to-face training [e.g., Dörrenbächer and Perels (2016)] that inherently limits the number of students who can participate. Therefore, Bellhäuser et al. (2016) developed a web-based training (WBT) to foster SRL strategies online. In their evaluation study, this WBT was demonstrated to have a positive effect on SRL knowledge, SRL behavior, and self-efficacy. However, the training also had a small detrimental effect on mathematics performance in an online mathematics preparation course. In a similar approach, Broadbent et al. (2020) tested the effect of a discipline-independent online training on SRL outcomes and found promising results, particularly when the online training was combined with a mobile-app based learning diary.

Both Bellhäuser et al. (2016) and Broadbent et al. (2020) followed an individual learning approach in which students acquired SRL strategies on their own through participation in the training. Thereby, students learned about the theoretical background of SRL strategies and were instructed to apply those strategies to a given example situation. In order to foster the application of those strategies in their daily lives, students additionally used a learning diary. Such diaries act as a prompt for SRL strategies by reminding students to formulate goals and to reflect on their learning behavior on a daily basis. However, both online trainings and learning diaries target individual students without taking advantage of the beneficial effects of collaborative learning (Johnson et al., 2000; Chen et al., 2018). Contact to fellow students that are also enrolled in the online training might help to keep up the motivation for following the training instructions. Additionally, peer students can provide valuable feedback on the learning process. The aim of the present study is therefore to augment the WBT applied by Bellhäuser et al. (2016) with a new peer feedback intervention that helps participants use the strategies from the WBT to improve their self-regulated learning as well as their performance.

### Process Model of Self-Regulated Learning

Our study is based on the process model of self-regulated learning by Schmitz and Wiese (2006), which is an adaptation of Zimmerman’s (2000) conception of self-regulation. According to this model, learning is a process that can be divided into three phases: pre-action, action, and post-action. These phases follow one another cyclically in every learning episode (i.e., one cycle of pre-action, action, and post-action phases such as homework on 1 day) and influencing the next learning episode (i.e., the next cycle of the phases such as homework on the next day) *via* a feedback loop. Every phase is characterized by a different set of tasks and challenges for the learner; therefore, different strategies and different competencies are required to achieve good learning results.

In the pre-action phase, learners establish goals according to the situation in which these students find themselves and the task with which the students are confronted. The next step is to deduce a plan to achieve these goals. If intrinsic and extrinsic motivation is not sufficient to initiate learning, self-motivation strategies serve as a further resource. In the action phase, learners operate with the actual learning content. Here, cognitive learning strategies (such as elaboration) and meta-cognitive learning strategies (such as monitoring) are crucial to learning success. Further, learners must utilize volitional strategies when observing a decrease in motivation to avoid procrastination. In the post-action phase, learners reflect on their learning episode and determine their level of satisfaction with their performance. For this purpose, learning goals are compared to actual achievement. The result of this comparison triggers the next pre-action phase in which learners establish new learning goals or modify unfinished goals.

### Fostering Self-Regulated Learning With Web-Based Training

The process model of SRL (Schmitz and Wiese, 2006) has been the foundation for many training interventions intended to foster SRL (Perels et al., 2005, 2009; Schmitz and Wiese, 2006; Leidinger and Perels, 2012; Werth et al., 2012; Dörrenbächer and Perels, 2016; Beek et al., 2020). Although those trainings differ in terms of the target groups, focus, and success, in all trainings, a human trainer conducts three or more face-to-face training sessions of approximately 2 h with a group of up to 30 participants. The effects of such trainings have been shown to be substantial not only in terms of improved self-reported learning behavior but also in increased performance (Dignath and Büttner, 2008; Benz, 2010). The disadvantages of face-to-face training, however, are that participants cannot flexibly choose when and where to attend training sessions and that trainers must restrict the number of participants in each training. For research purposes, another disadvantage is that sessions of face-to-face training are never absolutely identical on different occasions. Often because of time constraints, different persons conduct the trainings, leading to different effects. Even in studies in which only one person was the trainer, that person may have varied the exact wording of explanations from one training group to the next. Finally, with different participants in every training group, the quantity and quality of contributions by participants may also vary greatly.

Bellhäuser et al. (2016) therefore developed a web-based training that can be attended by virtually unlimited numbers of participants who are free to choose the time and location for their training. The WBT comprises three lessons of approximately 90 min each. The first lesson (“Before Learning”) focuses on the pre-action phase and covers goal-setting and time management. Lesson 2 (“During Learning”) addresses the action phase and covers volition, cognitive learning strategies and metacognitive learning strategies. The third lesson (“After Learning”) highlights the post-action phase and covers attribution and reflection. Each lesson utilizes videos, presentations, tests, exercises, and group discussions in an online forum.

The WBT was evaluated in the context of an online mathematics preparation course in which prospective students prepared themselves for their first university term in mathematically oriented fields of study (computer science, civil engineering, mechanical engineering, or mathematics). The preparation course occurred during the last four weeks before the university term began; covered mathematical knowledge from all school grades; and provided learners with definitions, arguments, examples, assignments, and visualizations. Because the preparation course was conducted completely online (created with the learning management system *Moodle*), no face-to-face instruction occurred. The preparation course took four weeks, during which all participants had the freedom to decide for themselves what to learn, when to learn, and how to learn.

In a randomized experimental design, Bellhäuser et al. (2016) investigated the effects of the WBT on SRL knowledge, self-regulated learning, self-efficacy, and mathematics performance. The intervention was deemed successful in conveying declarative knowledge regarding SRL, increasing self-efficacy, and improving self-reported SRL behavior. However, the results indicated a detrimental effect on participants’ mathematics performance. The authors discussed several possible explanations for this undesirable finding. The WBT required a certain amount of time that participants did not invest in the actual learning task (i.e., the preparation course). Furthermore, according to Siegler’s (2007) overlapping waves model, the acquisition of new strategies can impair performance in the short term, with beneficial effects appearing only in the long term. Finally, flaws in the mathematics test may have contributed to the decrease in mathematics performance. No matter how convincing these arguments may appear, an intervention with negative effects on performance is not satisfactory for practical use, and improvements in the training are therefore highly desirable.

Beek et al. (2019) applied the same WBT and compared its effects to a regular face-to-face training. They found equally high satisfaction with the two approaches and positive effects on subjective and objective learning outcomes for both presentation modes, thereby showing that web-based trainings can be feasible SRL interventions.

In a recent replication study, Broadbent et al. (2020) followed a similar approach, with the main differences that they implemented a discipline-independent online training [compared to the discipline-specific training from Bellhäuser et al. (2016)] and that they used mobile-app based diaries [compared to the browser-based application by Bellhäuser et al. (2016)]. The results confirmed that the online training had a positive effect on SRL and that a pure diary condition (without access to the online training) did not improve students’ SRL. The combined intervention condition outperformed both the pure training and the pure diary condition. However, no measures of actual performance were assessed in the study.

### Learning Diary Interventions

Learning diaries are a different approach for fostering SRL. Here, students are not instructed explicitly on SRL strategies; instead, they report their learning behavior in a short systematic collection of both open and closed questionnaire items. There are several mechanisms through which learning diaries are supposed to improve learning behavior. First, they are used to prompt SRL behavior daily (e.g., by asking questions such as “What are your learning goals for today?” in the morning or “How successful was your learning day?” in the evening), thereby acting as an external cue or reminder (Fabriz et al., 2014). This is particularly helpful because diaries are a method to reach students in their actual learning environment and not in an artificial situation. Second, learning diaries foster self-monitoring, drawing students’ attention to their own learning behavior (Schmitz and Perels, 2011). This is a necessary step toward critically reflecting whether one’s learning strategies are successful or need to be adjusted. Third, digital learning diaries can provide feedback on the learning process. By integrating interactive elements, students can be supported with graphical feedback about their learning behavior [e.g., the trajectory of procrastination: Wäschle et al. (2014)], about the status of their learning tasks (Neitzel et al., 2017), or even provide direct strategy instructions (Loeffler et al., 2019).

Multiple studies have shown that keeping such a diary over a certain time span (in many cases several weeks) can lead to improvements in SRL (Ewijk et al., 2015; Dörrenbächer and Perels, 2016; Loeffler et al., 2019). However, as there are also unsuccessful examples in the literature (Bellhäuser et al., 2016; Broadbent et al., 2020), it still remains unclear which circumstances are necessary for learning diaries to exhibit positive effects.

### Peer Feedback Interventions

In the evaluation forms, participants in the study by Bellhäuser et al. (2016) described bulletin boards in the WBT to be less helpful than elements of instruction such as videos and presentations. This response was surprising because as Davies and Graff (2005) stated, online discussions are expected to promote learning and performance. One possible explanation may be that participants did not know their peers on the bulletin boards and therefore did not have sufficient trust in their peers to share the details of their learning difficulties. Trust among members of virtual communities has been shown to be essential in the exchange of information (Ridings et al., 2002). Grouping participants into smaller peer groups (Wheelan, 2009) with a common interest such as a certain field of study (Ziegler and Golbeck, 2007) and the personal introduction of each participant (Rusman et al., 2009) can reduce anonymity and increase trust.

Peer feedback refers to “a communication process through which learners enter into dialogues related to performance and standards” (Liu and Carless, 2006). It involves at least two students that act as feedback giver and feedback receiver, with the feedback typically including both an assessment of the peer’s competency (feed-back) and a recommendation on how to proceed (feed-forward) (Hattie and Timperley, 2007). A recent meta-analysis (Huisman et al., 2019) demonstrated a rather large positive effect of receiving peer feedback on performance in academic writing tasks. Beneficial effects have further been shown for academic self-concept (Simonsmeier et al., 2020) and in other domains, such as language teaching, peer feedback has been shown to be successful in fostering affect and performance (Nelson and Schunn, 2009; Gielen et al., 2010). But not only the feedback receiver can profit from peer feedback: Zong et al. (2021) showed that feedback givers benefit even more than receivers. This might be the case because feedback givers need to reflect on the learning goals and the evaluation criteria as well as consider alternative solutions to a given task, all of which are learning strategies toward a deeper understanding of the topic (Bürgermeister et al., 2021). While peer feedback is often applied in situations where teachers cannot provide feedback themselves (e.g., in large courses), it should not necessarily be regarded as the second best solution. Huisman et al. (2019) found that peer feedback and teacher feedback lead to comparable achievements.

However, prior research has applied peer feedback only in the context of subject-specific academic tasks. To the best of our knowledge, there have been no attempts to foster non-specific SRL strategies by means of peer feedback. Given the known positive effects of teacher feedback on students’ self-regulated learning strategies (Azevedo et al., 2007), we expect peer feedback to be beneficial for both feedback receivers as well as feedback givers. Particularly in the context of a web-based SRL training that students work through individually, peer feedback groups might also help by reducing the feeling of loneliness, thereby increasing the motivation to complete the training.

### Research Questions

In the present study, we examined the effects of three different interventions designed to foster self-regulated learning. Prospective university students in an online mathematics preparation course were assigned to one of four experimental conditions: Group D (diary), Group TD (training + diary), Group TDP (training + diary + peer feedback group), and Group C (control). We expected each of the interventions to have positive effects on SRL knowledge, self-reported SRL behavior, self-efficacy, learning behavior (as measured by log file data) and mathematics performance.

Hypothesis 1 covered the positive effects of the learning diary. Because of the reactivity effect (Korotitsch and Nelson-Gray, 1999), we expected the diary to have a positive effect on SRL behavior (H1a), self-efficacy (H1b), mathematics performance (H1c), and time investment (H1d). These effects should result in greater gains for Group D than for Group C. However, we expected no effect on SRL knowledge because SRL strategies were not taught explicitly in the diary.

Hypothesis 2 covered the positive effects of the web-based training. By explicitly explaining SRL strategies and helping participants test the strategies personally (Bellhäuser et al., 2016), we expected the training to increase knowledge regarding SRL (H2a), thereby improving SRL behavior (H2b) and self-efficacy (H2c), which should result in increased mathematics performance (H2d). We also expected an increased time investment in the preparation course (H2e). The effects should be visible in the comparison between Group D and Group TD, with the latter achieving higher gains.

Hypothesis 3 covered the positive effects of the peer group interventions. Because students were deepening the content of the training and affiliating with peers, we expected statistically significant gains in SRL behavior (H3a), self-efficacy (H3b), mathematics performance (H3c), and time investment (H3d). These effects should exceed the gains of Group TD. No effect on SRL knowledge was expected.

## Materials and Methods

### Participants

We recruited 289 prospective students from an online mathematics preparation course at a technical university in Germany. The mean age was 19.8 years (SD = 1.48). Because participants were enrolled in mathematically oriented fields of study (computer science, civil engineering, mechanical engineering, or mathematics), the sample was predominantly male, comprising 233 male and 56 female students. We assigned participants randomly to one of four experimental conditions: Group D (Diary) kept a learning diary throughout the preparation course. Group TD (Training + Diary) had access to web-based SRL training and kept a learning diary. Participants in Group TDP (Training + Diary + Peer feedback group) also kept a diary and attended the web-based SRL training. In addition, members of Group TDP were placed in groups of five students each; these groups worked on additional SRL tasks that included peer feedback. Participants in control Group C did not have access to the training or the diary, nor were they placed into peer feedback groups. The randomized assignment controlled for gender and field of study by dividing the sample into eight subpopulations (2 gender × 4 fields of study, e.g., female mechanical engineers) and randomizing within each subpopulation separately. We expected more dropouts in Groups TD and TDC because of the higher workload and therefore assigned disproportionally more participants to these groups.

Complete data were obtained for 170 participants (134 male): 45 in group TDP (34 male), 45 in Group TD (37 male), 36 in Group D (29 male), and 44 in Group C (34 male). Because of the high dropout rate (41.2%), we investigated differences between participants and dropouts. Analyses revealed significantly lower scores in conscientiousness and the mathematics test for dropouts but no significant differences in demographic data (gender, age, school grades), SRL (including subscales), self-efficacy, extraversion, openness, agreeableness, or neuroticism.

### Procedure

The online mathematics preparation course is an e-learning course that covers the last 4 weeks before participants begin university lectures. The course is a voluntary option for students enrolled in mathematically oriented fields to prepare for course work, deepen school knowledge, and establish a common knowledge base among students (Bausch et al., 2014). The preparation course included six chapters (“Arithmetic,” “Powers,” “Functions,” “Higher Functions,” “Analysis,” and “Vectors”) with 52 mathematical topics, each of which comprised the following elements: diagnostic pre-test, overview, introduction to the domain, information, interpretation, application, typical mistakes, exercises, and diagnostic post-test. The preparation course was delivered in an online learning management system that involved no classroom instruction by tutors or teachers.

We chose this particular course because of its unique challenges regarding the self-regulation of the participants. The preparation course covers all topics that students are expected to be familiar with from school, resulting in a very large collection of instructions, examples, and self-tests. Working through this amount of material within 4 weeks therefore requires good time management skills. Further, there are no extrinsic factors to reinforce participation. The course was neither compulsory, nor were there grades or credit points for students to achieve. Finally, participants in this course were typically not well-prepared for such a learning environment. Most students came directly from school where they had little experience with self-regulated learning over long periods of several weeks, let alone on online learning platforms. Consequently, the mathematics preparation course was known for high dropout rates and low performance before we conducted our study.

After the mathematics course started, participants completed the online pre-test in the learning management system within the first three days, which comprised a demographic survey, an SRL knowledge test, a mathematics test, and several questionnaires that are discussed later. Depending on their experimental condition, participants had access to up to three separate interventions during the preparation course that were intended to foster SRL by different processes: a learning diary (prompting SRL strategies daily), a WBT on SRL (conveying SRL knowledge), and peer feedback groups (providing social support). The post-test was accessible online for three days after the end of the preparation course and comprised the SRL knowledge test, an SRL questionnaire, the mathematics test, and an evaluation sheet. As an incentive, all participants who completed both the pre- and post-tests were included in a lottery drawing (an electronic device and several monetary prizes).

### Interventions

#### Learning Diary

Groups D, TD, and TDP were requested to keep a learning diary throughout the preparation course. When filling in the diary, participants first decided whether they planned to learn on that day. If the students chose not to learn, the diary requested reasons and whether they planned to learn on the following day. Participants were further asked for their learning goals for the next learning day.

When participants chose to learn on a particular day, the students filled in two sections of the learning diary: one section to be completed before learning and one section to be completed after learning. Before learning, open-ended questions triggered goal-setting, planning, and self-motivation. Participants were requested to choose chapters from the preparation course to study on that day and set individual goals for those chapters (e.g., to solve all the problems and to get at least 70% of the problems correct). Learners were further asked which learning strategies they intended to apply and how much time they planned to invest. Closed questions were applied primarily for measuring purposes (e.g., motivation and well-being). Because this paper investigates the learning diary only as an intervention and not as a measurement instrument, the closed questions are not described in detail here.

The second section of the learning diary triggered reflection and goal-setting for the following day. Participants were asked which chapters they truly worked on and how much time they had invested in learning. By explicitly separating general time investment from effective learning time, participants critically reflected on their use of time. Learners were then requested to review the learning goals established in the first portion of the learning diary and judge the degree to which they had reached each goal. Further, students described which obstacles they had encountered during the day and how they planned to overcome such obstacles on the next learning day. For measuring purposes, participants rated their learning behavior on that day in closed questions (e.g., concentration, effort, and satisfaction). Participants made an average of *M* = 12.58 (*SD* = 4.92) learning diary entries over the course of the study.

#### Web-Based Training on Self-Regulated Learning

Groups TD and TDP had access to three lessons on self-regulated learning that were unlocked consecutively in 1-week intervals. Participants were asked to work through each lesson within a time frame of three days. Lessons were designed to take approximately 90 min. As described by Bellhäuser et al. (2016), the WBT imparts knowledge of the process model of self-regulated learning (Schmitz and Wiese, 2006) and utilizes videos, presentations, self-tests, exercises, and online bulletin boards to help participants transfer the knowledge to their daily learning routines.

Unlike Bellhäuser et al. (2016), we did not include animated videos. Instead, real-life videos were created by two amateur actors in a real classroom scenario, one actor acting as the trainer, the other actor acting as a participant in the training. Choosing human actors was intended to increase credibility and personalize the experience for the audience, thereby improving satisfaction with the WBT.

The first lesson, “Before Learning,” covered the pre-action phase, including chapters on goal-setting, planning, and time-management. Participants were advised to establish learning goals for the preparation course according to the SMART technique (Doran, 1981). After a presentation regarding time-management, participants reflected on their own time-management and discussed individual problems on a bulletin board. The last step was developing a learning plan for the entire four weeks of the preparation course, considering personal learning goals and time restrictions such as chores or hobbies.

The second lesson, “During Learning,” focused on the action phase, the chapters including volitional learning strategies (such as addressing distractions and avoiding procrastination) and cognitive and metacognitive learning strategies. A video introduced the concept of procrastination, and participants analyzed whether they were prone to delaying tasks. To avoid distractions in the future, participants were advised to switch off mobile phones and communication software on their computers before entering the preparation course. Self-motivation strategies (e.g., self-reward) were presented, and participants developed a personal motto for situations in which they may lack motivation to learn. Referring to examples from the preparation course, presentations explained how to use cognitive learning strategies (e.g., structuring, elaborating, and summarizing) and metacognitive learning strategies (particularly monitoring).

The third lesson, “After Learning,” addressed the post-action phase, including chapters on attribution, frame of reference, reflection, and motivation. A video exemplified different attribution styles in the face of failure. Participants were encouraged to identify personal but changeable causes to alter motivation. Similarly, an individual frame of reference was promoted: Instead of comparing oneself to other students, participants were instructed to focus on improving their own performance. In the chapter on reflection, a presentation explained how reflection can be applied on a short-term basis (e.g., whether one successfully solved a particular mathematical problem), on a medium-term basis (e.g., whether one was satisfied with today’s learning progress), and on a long-term basis (e.g., whether one would approach future examinations in a different manner). Participants were instructed to review their learning goals from Lesson 1 and to reflect on necessary adjustments for the remaining days of the preparation course. In the last chapter on motivation, implementation intentions (Gollwitzer, 1999) were presented as a strategy to increase motivation. After a summary of the process model of self-regulated learning, the training ended with participants writing a letter to their future selves regarding what they planned to change in their learning behavior.

In the final evaluation of the study, we asked participants to which degree they followed the instructions in the web-based training. Mean compliance was *M* = 82.18% (*SD* = 15.03%).

#### Peer Feedback Intervention

Participants in Group TDP were assigned to peer feedback groups of five persons each. Although group assignments were random, when possible, group members were chosen from the same field of study (e.g., five civil engineers). Peer feedback groups were able to communicate on a separate bulletin board on which discussion topics were suggested. Beginning with a welcome message, participants were encouraged to get to know their peers by creating quiz questions about themselves, posting them on the bulletin board, and guessing the right answers to their peers’ quiz questions. After each lesson of the WBT, a group task referring to the current lesson was posted; this task was meant to be solved collaboratively. Lesson 1 was followed by the group task of sharing students’ individual time schedules and commenting on their peers’ plans (peer feedback Task 1). After Lesson 2, participants were asked to discuss the cognitive learning strategies taught in the lesson and how to apply those strategies to the mathematical chapters (peer feedback Task 2). The group task for Lesson 3 was to reflect on their time management in the preparation course to date and to adjust their learning goals if necessary (peer feedback Task 3). Although discussion regarding the content of the mathematical preparation course was not forbidden, the instructional topics were only related to strategies of self-regulated learning behavior. Inspection of the bulletin boards revealed that participants focused on the instructed group tasks.

All instructions for the discussions were also presented in videos. When members of a group did not participate in the group discussion, the experimenters reminded and encouraged participants to engage; however, no pressure was applied. In the final evaluation, participants rated their personal active engagement in the peer feedback groups on a six-point Likert scale. Mean active engagement was *M* = 3.18 (*SD* = 1.57).

### Instruments

#### Self-Regulated Learning Questionnaire

The self-regulated learning questionnaire comprised 26 items with seven subscales. The overall score had a Cronbach’s α of .85. The sub-scales were goal-setting (four items, Cronbach’s α = 0.66, e.g., “*I choose my goals so that they are a challenge for me.*”), planning (four items, Cronbach’s α = 0.63, e.g., “*I write down all important tasks and appointments.*”), self-motivation (three items, Cronbach’s α = 0.71, e.g., “*I recall my past achievements to motivate myself for difficult tasks.*”), volition (four items, Cronbach’s α = 0.71, e.g., “*I can modify my mood so that I find everything easier.*”), elaboration (three items, Cronbach’s α = 0.71, e.g., “*When reading, I try to connect the things I am reading about with what I already know.*”), metacognition (four items, Cronbach’s α = 0.64, e.g., “*I regularly think about my learning behavior.*”), and reflection (four items, Cronbach’s α = 0.78, e.g., “*At the end of a day, I ask myself whether I am satisfied with my performance.*”); all subscales were determined to be sufficiently reliable. The questionnaire was developed in the context of prior studies to match the content of the WBT. Most items were newly created, except for three items from the LIST (Wild and Schiefele, 1994) and six items from the VCQ (Kuhl and Fuhrmann, 1998).

#### Self-Regulated Learning Knowledge Test

The SRL knowledge test included 20 multiple-choice items (Cronbach’s α = 0.81). Participants were required to choose one of four possible answers: One choice was the correct answer and three were distractors. Calculating the number of correct answers resulted in a total score of 0 to 20 points. The questions concerned constructs that were explained in the WBT, e.g., “According to the process model of self-regulated learning, what should you do in the pre-action phase? (a) set goals (right answer), (b) concentrate (distractor), (c) reflect (distractor), (d) relax (distractor).”

#### Self-Efficacy

We applied the Generalized Self-Efficacy Scale (Schwarzer and Jerusalem, 1999), which comprises ten items (Cronbach’s α = 0.78, e.g., “*I can always manage to solve difficult problems if I try hard enough.*”).

#### Mathematics Test

The mathematics test, comprising 52 problems (Cronbach’s α = 0.84), was created by mathematicians who were responsible for the preparation course. Each problem addressed one of the chapters in the course. In two parallel versions (before and after the mathematics course), participants were allotted 60 min; the time investment was measured to identify lack of engagement in the test. With one point for each correct solution, the *mathematics overall score* ranged from 0 to 52.

Additionally, participants were requested to choose ten chapters to particularly focus on, according to their individual needs. The corresponding ten problems on the mathematics test were calculated to determine the *mathematics focus score* (ranging from 0 to 10).

#### Time Investment

We collected logfile data from the learning platform *Moodle* on which the mathematics course was hosted. Each click on the platform created a logfile entry containing the username, time and date, and the content being clicked on. Learning sessions were defined as a sequence of logfiles without interruptions of more than 30 min. For each participant, we calculated the duration of each learning session and added these durations as a measure of time investment.

## Results

### Screening Procedure

We compared the time investment on the mathematics pre- and post-tests to identify participants who did not apply sufficient effort on the post-test. The rationale behind this comparison was that participants may have simply opened the mathematics test to fulfill the criteria for the lottery drawing. We therefore excluded participants who spent 20% less time on the mathematics post-test than the same participants spent on the mathematics pre-test, resulting in a sample of 136 participants.

Descriptive statistics for all dependent variables in the final sample are shown in Table 1. For all dependent variables, we calculated one-way ANOVAs with the pre-test data in order to check whether starting conditions between the four experimental groups differed significantly. This was not the case for any of the variables: SRL knowledge test [*F*_(3,132)_ = 0.58; *p* = 0.631]; self-efficacy [*F*_(3,132)_ = 0.62; *p* = 0.607]; SRL overall score [*F*_(3,132)_ = 0.95; *p* = 0.420]; Mathematics overall score [*F*_(3,132)_ = 0.31; *p* = 0.817]; Mathematics focus score [*F*_(3,132)_ = 1.18; *p* = 0.320].

**TABLE 1:** Mean and standard deviation for each experimental group for self-regulated learning (SRL) knowledge, self-efficacy, overall SRL score, SRL subscales, mathematics overall score, and mathematics focus score on pre- and post-tests.

	Group C (*n* = 34)	Group D (n = 28)	Group TD (*n* = 40)	Group TDP (*n* = 34)
	
	*M* (SD)	*M* (SD)	*M* (SD)	*M* (SD)
SRL knowledge test				
Pre-test	3.34 (1.76)	3.50 (1.59)	3.74 (1.55)	3.81 (1.79)
Post-test	3.28 (2.07)	3.54 (2.10)	7.69 (1.35)	8.43 (0.83)
Self-efficacy				
Pre-test	4.23 (0.76)	4.16 (0.74)	4.04 (0.69)	4.03 (0.78)
Post-test	4.10 (0.69)	4.25 (0.77)	4.23 (0.69)	4.31 (0.69)
SRL overall score				
Pre-test	3.52 (0.56)	3.72 (0.59)	3.50 (0.61)	3.65 (0.66)
Post-test	3.52 (0.65)	3.65 (0.68)	3.81 (0.67)	4.17 (0.67)
SRL goal-setting				
Pre-test	4.78 (0.76)	4.90 (0.75)	4.54 (0.83)	4.79 (0.79)
Post-test	4.50 (0.88)	4.79 (0.73)	4.56 (0.74)	4.92 (0.61)
SRL planning				
Pre-test	3.48 (0.97)	3.38 (1.09)	3.42 (1.17)	3.60 (0.84)
Post-test	3.54 (0.93)	3.66 (1.09)	3.98 (1.02)	4.40 (0.79)
SRL self-motivation				
Pre-test	4.31 (1.28)	4.44 (1.04)	4.18 (1.02)	4.05 (1.24)
Post-test	4.28 (1.25)	4.07 (1.09)	4.50 (0.93)	4.55 (0.92)
SRL volition				
Pre-test	3.21 (0.79)	3.62 (0.98)	3.24 (0.85)	3.40 (0.93)
Post-test	3.32 (0.84)	3.35 (1.06)	3.57 (0.95)	3.88 (1.08)
SRL elaboration				
Pre-test	4.38 (1.00)	4.45 (1.00)	4.03 (1.06)	4.40 (0.98)
Post-test	4.06 (0.97)	4.26 (0.97)	4.23 (0.83)	4.64 (0.91)
SRL metacognition				
Pre-test	2.10 (0.64)	2.24 (0.82)	2.23 (0.71)	2.31 (0.93)
Post-test	2.29 (0.74)	2.42 (0.84)	2.67 (0.75)	3.19 (1.20)
SRL reflection				
Pre-test	2.79 (1.09)	3.35 (0.98)	3.16 (1.00)	3.29 (1.21)
Post-test	2.96 (1.02)	3.23 (0.99)	3.44 (1.04)	3.82 (1.06)
Mathematics overall score				
Pre-test	19.48 (6.62)	19.93 (7.40)	19.89 (5.87)	18.63 (5.45)
Post-test	19.08 (7.41)	21.16 (8.10)	21.66 (5.91)	20.69 (7.00)
Mathematics focus score				
Pre-test	2.95 (1.51)	2.44 (1.57)	2.64 (1.61)	2.27 (1.53)
Post-test	2.98 (1.92)	3.18 (1.94)	3.20 (1.71)	3.50 (1.62)

### Evaluation of Training Effects

**FIGURE 1:**
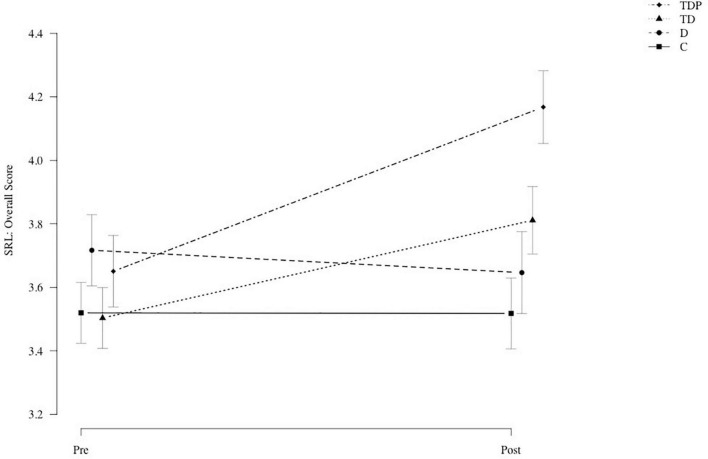
Self-regulated learning (SRL) overall scores on pre- and post-tests for Groups C (control group), D (diary), TD (training + diary), and TDP (training + diary + peer feedback intervention).

We calculated three separate repeated-measures MANOVAs with group and time as the independent variables and different sets of dependent variables. In the first MANOVA, we entered SRL knowledge, self-efficacy, mathematics overall score, and SRL overall score as the dependent variables. The results showed a statistically significant effect of the group [Pillai’s trace = 0.51, *F*_(3,132)_ = 6.70; *p* < 0.001], a statistically significant main effect of time [Pillai’s trace = 0.66, *F*_(1,132)_ = 61.78; *p* < 0.001], and a statistically significant interaction between the factors [Pillai’s trace = 0.71, *F*_(3,132)_ = 10.19; *p* < 0.001], justifying running univariate ANOVAs for the four dependent variables. As seen in Table 2, SRL knowledge, self-efficacy and the SRL overall score showed statistically significant interaction effects in the hypothesized direction, with Group TDP showing the most prominent gains among treatment groups and Group C showing either constant levels or even negative developments. Figure 1 depicts the increases in the SRL overall score for all four experimental groups. The interaction effect for the mathematics overall score, however, marginally missed the level of statistical significance although descriptive statistics indicated the hypothesized direction.

**TABLE 2:** Univariate repeated-measures ANOVAs for self-regulated learning (SRL) knowledge, self-efficacy, overall SRL score, SRL subscales, mathematics overall score, and mathematics focus score on pre- and post-tests.

	Main effect group				Main effect time				Interaction effect			
	*df*	*F*	*p*	η*^2^p*	*df*	*F*	*p*	η*^2^p*	*df*	*F*	*p*	η*^2^p*
SRL knowledge test	3, 132	38.20	<0.001	0.46	1, 132	206.34	<0.001	0.40	3, 132	59.23	<0.001	0.34
Self-efficacy	3, 132	0.06	0.978	0.00	1, 132	9.44	0.003	0.06	3, 132	5.914	<0.001	0.11
SRL overall score	3, 132	2.49	0.063	0.05	1, 132	29.40	<0.001	0.15	3, 132	12.55	<0.001	0.19
SRL goal-setting	3, 132	1.67	0.177	0.04	1, 132	0.99	0.322	0.01	3, 132	2.34	0.076	0.05
SRL planning	3, 132	2.03	0.112	0.04	1, 132	43.70	<0.001	0.23	3, 132	5.94	<0.001	0.09
SRL self-motivation	3, 132	0.04	0.989	0.00	1, 132	3.479	0.064	0.01	3, 132	6.79	<0.001	0.13
SRL volition	3, 132	1.75	0.322	0.03	1, 132	6.69	0.011	0.04	3, 132	4.58	0.004	0.09
SRL elaboration	3, 132	1.34	0.263	0.03	1, 132	0.01	0.973	0.00	3, 132	3.88	0.011	0.08
SRL metacognition	3, 132	3.37	0.020	0.07	1, 132	41.07	<0.001	0.22	3, 132	5.66	0.001	0.09
SRL reflection	3, 132	3.06	0.030	0.07	1, 132	7.73	0.006	0.05	3, 132	2.37	0.074	0.05
Mathematics overall score	3, 132	0.44	0.727	0.01	1, 132	11.50	<0.001	0.08	3, 132	2.50	0.062	0.05
Mathematics focus score	3, 132	0.06	0.978	0.00	1, 132	17.31	<0.001	0.11	3, 132	2.69	0.049	0.05

**FIGURE 2:**
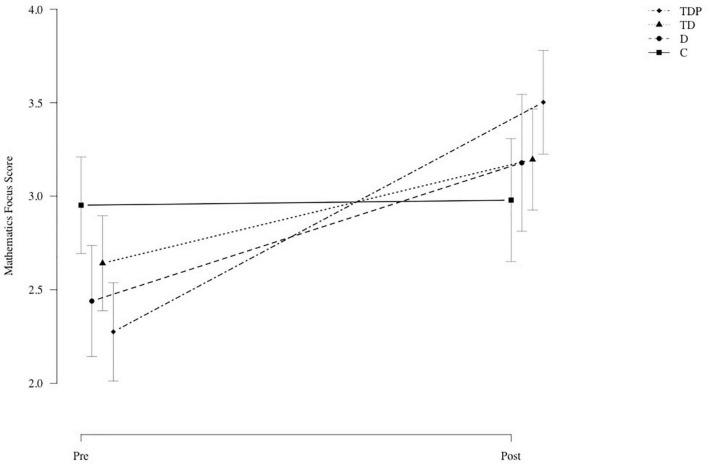
Mathematics focus scores on pre- and post-tests for Groups C (control group), D (diary), TD (training + diary), and TDP (training + diary + peer feedback intervention).

For the second MANOVA, we replaced the mathematics overall score with the mathematics focus score, which was calculated individually for the ten chapters that each participant personally chose as the most important. The rationale was that improved SRL competency after the intervention may lead to a stronger focus on personal goals rather than improved performance in all chapters (including those chapters outside of individual focus). Because the mathematics focus score was calculated only on chapters that participants chose to be personal goals, it appears reasonable that gains were manifested in this score rather than the overall score. Again, the MANOVA showed a statistically significant main effect of the group [Pillai’s trace = 0.51, *F*_(3,132)_ = 6.66; *p* < 0.001], a statistically significant main effect of time [Pillai’s trace = 0.66, *F*_(1,132)_ = 62.22; *p* < 0.001], and a statistically significant interaction of the two factors [Pillai’s trace = 0.73, *F*_(3,132)_ = 10.49; *p* < 0.001]. The univariate ANOVA for the mathematics focus score in fact revealed a statistically significant interaction effect between group and time (see Table 2). Gains for the four experimental groups in the mathematics focus score are presented in Figure 2.

To investigate the group differences in depth, we calculated contrasts for the selection of dependent variables used in the second MANOVA. We tested whether the gains of the four experimental groups (e.g., mathematics focus score for Group TD in the post-test minus mathematics focus score for Group TD in the pre-test) differed from zero in a statistically significant manner.

As seen in Table 3, Group TD showed statistically significant increases in SRL knowledge (β = 3.95; *p* < 0.001), in the SRL overall score (β = 0.31; *p* < 0.001) and in self-efficacy (β = 0.20; *p* = 0.04) but not in mathematics scores. Similarly, for Group TDP, the increases in SRL knowledge (β = 4.61; *p* < 0.001), in the SRL overall score (β = 0.52; *p* < 0.001) and in self-efficacy (β = 0.28; *p* < 0.01) were determined to be statistically significant. By contrast to Group TD, Group TDP showed statistically significant increases in the mathematics focus score (β = 1.23; *p* < 0.001). Groups C and D showed no statistically significant increases in any dependent variable.

**TABLE 3:** Planned contrasts: gains of the four experimental groups from pre-test to post-test.

	Group C (*N* = 34)	Group D (*N* = 28)	Group TD (*N* = 40)	Group TDP (*N* = 34)
	
	β (SE)	β (SE)	β (SE)	β (SE)
SRL knowledge test	−0.06 (0.32)	0.04 (0.35)	3.95 (0.30)***	4.62 (0.32)***
Self−Efficacy	−0.13 (0.07)	0.09 (0.08)	0.20 (0.07)*	0.28 (0.07)**
SRL overall score	0.00 (0.08)	−0.07 (0.08)	0.31 (0.07)***	0.52 (0.08)***
SRL goal−setting	−0.28 (0.11)	−0.12 (0.13)	0.02 (0.11)	0.13 (0.11)
SRL planning	0.06 (0.13)	0.29 (0.15)	0.55 (0.12)***	0.80 (0.13)***
SRL self−motivation	−0.04 (0.14)	−0.37 (0.16)	0.32 (0.13)	0.50 (0.14)**
SRL volition	0.12 (0.15)	−0.28 (0.16)	0.33 (0.13)	0.48 (0.15)*
SRL elaboration	−0.32 (0.14)	−0.19 (0.16)	0.20 (0.13)	0.23 (0.14)
SRL metacognition	0.19 (0.14)	0.18 (0.15)	0.44 (0.13)**	0.88 (0.14)***
SRL reflection	0.16 (0.16)	−0.12 (0.18)	0.28 (0.15)	0.52 (0.16)*
Mathematics focus score	0.03 (0.30)	0.74 (0.33)	0.56 (0.28)	1.23 (0.30)***

*p < 0.05; **p < 0.01; ***p < 0.001.

In the third MANOVA, we examined the influence of the interventions on the SRL subscales goal-setting, planning, self-motivation, volition, elaboration, metacognition, and reflection. Here as well, we observed a statistically significant main effect of group [Pillai’s trace = 0.62, *F*_(3,132)_ = 3.23; *p* < 0.001], a statistically significant main effect of time [Pillai’s trace = 0.70, *F*_(1,132)_ = 28.15; *p* < 0.001], and a statistically significant interaction between the two factors [Pillai’s trace = 0.85, *F*_(3,132)_ = 4.98; *p* < 0.001]. The results of the following univariate ANOVAs are presented in Table 2. The subscales planning, self-motivation, volition, elaboration, and metacognition all revealed statistically significant interaction effects consistent with our hypotheses, with Group TDP outperforming the other two intervention groups and control Group C showing no positive or negative trends. For the subscales goal-setting and reflection, the interaction effects missed statistical significance although descriptive data indicated the hypothesized direction.

Again, we calculated contrasts for the selection of the dependent variables used in the third MANOVA to investigate gains of the four experimental groups (see Table 3). Although Group C and Group D showed no statistically significant increases in any of the SRL subscales, Group TD showed statistically significant increases in planning (β = 0.55; *p* < 0.001) and in metacognition (β = 0.44; *p* < 0.01). Group TDP also showed statistically significant increases in planning (β = 0.80; *p* < 0.001) and in metacognition (β = 0.88; *p* < 0.001); in addition, Group TDP showed statistically significant increases in self-motivation (β = 0.50; *p* < 0.01), volition (β = 0.48; *p* = 0.01), and reflection (β = 0.52; *p* = 0.02). However, gains in goal-setting and elaboration remained statistically non-significant for Group TDP.

Using a one-way ANOVA, we analyzed time investment in the preparation course measured by log files. Because there was no pre-test score for this measure, we could not include this variable in the MANOVA models described above. The differences between group means (Group C: *M* = 21.03 h, *SD* = 17.56; Group D: *M* = 28.23 h, *SD* = 14.13; Group TD: *M* = 29.32 h, *SD* = 17.79; Group TDP: *M* = 33.56 h, *SD* = 18.87) were determined to be significant (*F*(3, 132) = 3.08; *p* = 0.030; $\eta_{p}^{2}$ = 0.06). Contrast analyses revealed that differences between adjacent Groups C and D (*p* < 0.01), D and TD (*p* = 0.02), and TDP and TD (*p* = 0.03) all were significant. Notably, the log files only reflected time spent on the mathematics platform; the files did not include time spent with the three interventions learning diary, WBT, and peer feedback groups.

## Discussion

The present study investigated the effects of three separate interventions that all proposed to foster self-regulated learning in an e-learning environment. A sample of 136 prospective students (after dropout and data cleansing) participated in an online mathematics preparation course for four weeks before beginning their first university semester in mathematically oriented fields. Participants were randomized into one of four experimental groups that had access to either a learning diary (Group D), a combination of a diary and web-based self-regulation training (Group TD), a combination of a diary, web-based training and a peer-feedback intervention (Group TDP), or none of the interventions (control Group C). We measured the effects on an SRL knowledge test, an SRL questionnaire, and a self-efficacy questionnaire. To assess mathematical performance, we administered a mathematics test that covered all the chapters from the preparation course. In addition to the overall score for this test, a focus score was calculated for a selection of mathematical problems that each participant chose to be particularly important to that participant personally. Furthermore, log files from the mathematics learning platform were analyzed with regard to time investment.

We conducted a series of analyses that began on a rather broad top level (MANOVA for all dependent variables), followed by a more detailed middle level (separate ANOVAs for each dependent variable), and ending on a quite specific low level (separate contrasts for gains of each experimental group in each dependent variable). Lower levels of analyses only occurred if significant results on the respective higher level warranted deeper inspection of the effects. All top-level MANOVAs showed significant interaction effects, indicating that different developments in the four groups occurred in at least some of the dependent variables. The following ANOVAs revealed statistically significant interaction effects for all dependent variables, except for the mathematics overall score and the SRL subscales goal-setting and reflection. Because these findings did not provide information regarding the exact groups between which statistically significant differences occurred, we relied primarily on the contrast analyses to decide whether to accept or reject our hypotheses.

In Hypothesis 1, we postulated positive effects of the learning diary on self-reported SRL behavior, self-efficacy, mathematics performance, and time investment. None of the increases reached statistical significance. We only observed a greater time investment for the diary group compared with the control group. In the context of the present preparation course, this result may be regarded as desirable. Although in other learning scenarios, an increased time investment is not necessarily beneficial, a mean time investment of only 21 h in the control group cannot possibly be sufficient to review all chapters of the preparation course when the responsible lecturers estimated a duration of 4 weeks of full-time work. A mean increase of seven hours in Group D, although desirable, is not satisfactory.

We therefore reject the first hypothesis. The learning diary used in the present study clearly did not provide substantial help to participants. This result matches findings from Bellhäuser et al. (2016), who observed no positive effects of a learning diary in a setting comparable to the present study. Perhaps the diary should have been accompanied by a tutorial explaining the potential benefits of learning diaries as demonstrated in other studies (Korotitsch and Nelson-Gray, 1999; Schmitz and Perels, 2011).

In Hypothesis 2, we postulated positive effects of the web-based self-regulation training on declarative SRL knowledge, self-reported SRL behavior, self-efficacy, and mathematics performance, exceeding the effects of the diary-only intervention. As expected, both groups with access to the web-based training increased declarative knowledge regarding SRL. This result may be regarded as a manipulation check that was positive. For the SRL questionnaire, we observed statistically significant increases in Group TD that were not present in Group D, indicating that the additional WBT was responsible for this improvement. Investigating the seven subscales of the SRL questionnaire provided even more detailed insights: Group TD outperformed Group D on the subscales planning and metacognition. Clearly, the WBT was particularly successful in conveying these contents. Furthermore, we observed a statistically significant increase in self-efficacy for Group TD although less prominent than the gains on the SRL questionnaire. For mathematics performance, we did not observe gains in Group TD beyond the general positive main effect for time that was observed for all experimental groups. Concerning time investment, we observed a statistically significant difference between Groups TD and D (and therefore necessarily also between TD and C).

Combining the results of the web-based training on SRL, we concluded that our hypothesis can be accepted with one exception: The WBT helped participants improve their SRL knowledge, their SRL behavior (predominantly in the domains of planning and metacognition), their self-efficacy, and their time investment but not their mathematics performance. Comparing these results to Bellhäuser et al. (2016) leads us to believe that the WBT has been substantially improved in the present study because the prior study revealed small, yet negative effects of the WBT on mathematics performance.

Hypothesis 3 postulated positive effects of the peer feedback intervention groups on self-reported SRL behavior, self-efficacy, mathematics performance, and time investment, above and beyond the effects of the pure web-based training. We found significantly positive effects in most of the dependent variables for Group TDP that were either non-significant in Group TD (e.g., mathematics focus score or volition) or less pronounced (e.g., SRL overall score or self-efficacy).

As expected, the participants in Group TDP experienced increases in declarative SRL knowledge identical to the gains in Group TD. For self-reported SRL behavior, both the overall score and the subscales planning and metacognition showed gains, mirroring the results from Group TD and Group TDP. However, whereas Group TD experienced no statistically significant increases in any of the other subscales, Group TDP showed statistically significant improvements in self-motivation, volition, and reflection. The additional peer feedback intervention appears to have facilitated better use of the strategies concerning self-motivation, volition, and reflection taught in the WBT.

Because the peer feedback tasks involved discussions regarding the individual time schedule (Task 1 after Lesson 1 of the WBT), cognitive learning strategies (Task 2 after Lesson 2 of the WBT), and reflection on their progress to date (Task 3 after Lesson 3 of the WBT), we believe that all SRL subscales were targeted by the peer feedback intervention: Goal-setting and planning were addressed in peer feedback Task 1 and Task 3; self-motivation, volition, and reflection were primarily addressed in peer feedback Task 3; elaboration and metacognition were primarily addressed in peer feedback Task 2. We therefore deem it plausible that Group TDP showed greater gains than Group TD on most SRL subscales. Nevertheless, no statistically significant increases could be detected for the subscales goal-setting and elaboration. For goal-setting, this may be the result of a ceiling effect—this subscale showed the highest pre-intervention scores, leaving less room for improvements than the other subscales. In the case of elaboration, the rather general learning strategies taught in the WBT may not have been sufficiently adjusted to the exact context of the mathematics preparation course. The peer feedback following Task 2 (discussing the use of the learning strategies taught in the WBT) can clearly only improve elaboration (as measured by our questionnaire) if the strategies taught in the WBT in fact fit the needs of participants in the preparation course. For self-efficacy, we observed slightly higher gains in Group TDC compared with Group TD. However, this positive effect appears to be rather small.

In our first analysis, the effect on the mathematics performance remained below the level of statistical significance because we evaluated the mathematics overall score (including all problems from the mathematics test). When examining mathematics focus scores (including only those problems from chapters that participants chose as important to those participants personally) we observed statistically significant increases for Group TDP. However, this increase was rather small and should not yet be regarded as strong empirical evidence. We assume that changes in self-regulated learning behavior need more time than the given 4 weeks in this study in order to have an impact on learning performance.

The mean time investment in Group TDP was 33 h, which is longer than time spent in the other groups but nevertheless still failed to meet the expectations of the responsible lecturers of the preparation course. However, voluntary mathematics preparation courses without face-to-face interaction with tutors and peers, particularly in the age group of approximately 20-year-olds, may have had little chance to convince participants to sacrifice more of their leisure time.

The results from the peer feedback intervention groups support Hypothesis 3: The combined intervention in Group TDP helped participants increase their declarative SRL knowledge, improve their SRL behavior (in all but two subscales), increase self-efficacy, increase their time investment, and improve their mathematics performance. Compared with the results of Bellhäuser et al. (2016), the supplementary peer feedback tasks appeared to substantially improve the quality of the intervention. Because the time span of the present study was only four weeks and the combined intervention only took a few hours (including all three lessons of the WBT, the corresponding peer feedback tasks, and the learning diary), we consider the combined intervention quite successful and efficient.

### Limitations

The major limitation of the present study concerns the sample of participants: Because the mathematics course serves to prepare students for mathematically oriented fields (computer science, civil engineering, mechanical engineering, and mathematics), our sample was predominantly male and may not be representative of students from other fields. The rather large dropout rate in our study exacerbates this issue. However, we could only identify statistically significant differences between dropouts and remaining participants in conscientiousness and the mathematics test with the majority of the other variables showing no differences. The number of dropouts in our study, however, can be described as typical for the learning scenario: The voluntary online mathematics course took place before the regular university courses started and was not reinforced, controlled, or graded. The responsible lecturer reported dropout rates of up to 80% in the recent years. Therefore, dropout in our study might also have been due to a general dropout in the mathematics course.

Another limitation arises from our study design: We did not separate the three different interventions (diary, WBT, and peer feedback intervention) but rather chose a nested design that tested a selection of three different combinations against one another. This approach was selected partially because the peer feedback intervention tasks were inherently cumulative to the web-based training and would not have made sense in isolation. A completely balanced design with all eight combinations of interventions was therefore not feasible; the sample size within each cell could have been problematic as well. We opted to leave out a possible Group T (web-based training without diary or peer feedback intervention) because Bellhäuser et al. (2016) included such a condition in their design. However, we implemented instead the diary-only Group D, mostly to collect time-series data for participants without access to the WBT although the present paper does not include these analyses.

One concern regarding our study may be that improvements in the mathematics test across all experimental groups are relatively small. Of 53 possible points, the global mean was 19.5 on the pre-test and 20.7 on the post-test. Although this main effect of time did reach statistical significance, the effect did not meet expectations (similar to the manner in which the time investment of participants was not satisfying either). Part of this result may be attributed to the target group: The preparation course aimed at gaps in mathematics school knowledge, therefore strong students might have decided to never take the course in the first place. Further, the mathematics test perhaps was too difficult or that the allotted time was too restrictive. Also, the overall time investment was very low even in the experimental groups—students might simply have underestimated how much time they would need in order to complete the course. Another reason may be that participants were more motivated and concentrated more during the pre-test than the post-test, particularly because the test had no consequences for the students’ future field of study. Without the external pressure, the primary motivation for good performance may have been to evaluate one’s own knowledge and possibly compare oneself to future peers. Because the pre-test had previously provided crucial feedback evaluating current knowledge, when the time came for the post-test, some participants may have felt only the need to complete the test for the lottery—the self-evaluating aspect of the mathematics test may have been less important. Furthermore, allocating one uninterrupted hour for the mathematics test and trying to focus as much as possible on that test may have been easier for participants at the beginning of the preparation course (one month before beginning of the semester) than at the end of the course (a few days before the first lectures). Organizational problems such as moving to a different city or managing a household for the first time on one’s own possibly conflicted more with academic aspirations on the post-test than on the pre-test.

### Summary and Future Research

Our results indicated that the combined intervention comprising the learning diary, web-based training, and self-regulated learning with subsequent peer feedback intervention was the most successful, with beneficial effects on self-regulated learning, time investment, and self-efficacy. The effect on mathematics performance was only found for the focus score—a selection of personally relevant topics—and was only very small. However, it remains possible that the improved learning strategies had a delayed effect on performance. There are examples of SRL interventions in which positive effects were stronger in follow-up tests than immediately after the intervention [e.g., Stoeger et al. (2014)].

The combination of the learning diary and web-based training without peer feedback intervention was determined to have statistically significant yet slightly less pronounced effects on self-regulated learning, time investment, and self-efficacy but not on mathematics performance. Using a learning diary without supplementary interventions did not appear to improve self-regulated learning. However, as learning diaries can detect fluctuations in motivation (Bellhäuser et al., 2021), they still seem to be a promising intervention approach when developed further to provide adaptive situation-specific feedback (Loeffler et al., 2019).

Because WBT, once that training is created, can serve virtually unlimited numbers of participants, we advocate its application in educational settings in which large groups of students require support in their self-regulated learning, particularly in distance learning environments that prevent face-to-face training. The additional peer feedback intervention appears to be a useful supplement to WBT, and its organizational costs are comparably low: Participants were assembled into groups of five and were given a group discussion task after each of the three lessons of the WBT. These group discussions regarding their individual learning schedules, their learning strategies, and their progress in the preparation course appeared to substantially increase the beneficial effects of the WBT.

Future studies should investigate the mechanisms of the peer feedback intervention. The mere act of forming small groups could have increased motivation, particularly because the online preparation course may be experienced as a rather solitary task. Our choice of group discussion tasks was theoretically grounded in the process model of SRL (Schmitz and Wiese, 2006); however, it would be possible to create different group tasks to investigate the effects of the exact formulation of the task. In our study, participants did not receive instruction on how to give feedback. As shown by Gielen et al. (2010), explaining to students the criteria of good peer feedback can increase the effectiveness of peer feedback. Also, providing guidance for the assessment of peers’ performance (e.g., rubrics) can improve the quality of peer feedback (Bürgermeister et al., 2021). Finally, visualizations of the performance of relevant peers (e.g., sharing similar goals or prior knowledge) might enable students to develop a realistic estimate for their own goal setting (Konert et al., 2016).

A completely different yet certainly also promising approach would be to have learning groups discuss the actual learning content rather than their learning behavior on a meta-level. In the case of the online preparation course, members of a learning group could be asked to discuss their understanding of mathematical problems or even solve complex problems collectively. Possibly the best support for learners would be to combine group tasks that cover the actual learning content with a task that focuses on self-regulated learning.

Although the overall effect of the peer feedback intervention was convincing, not all groups benefitted to the same extent. It appears worthwhile to investigate the causes of inter-group differences. One approach may be to improve group formation by considering personality traits when determining the composition of groups (Bellhäuser et al., 2018; Müller et al., 2022). Also, technical expertise appears to be a key variable for virtual teams, and group composition should perhaps consider a minimum level of technical expertise for every team.

Another approach may be to provide more support for the teamwork process. In particular, asynchronous communication appears to be an issue (Durnell Crampton, 2002). Inactivity or delayed activity on virtual teams can lead to problems in communication; participants may require instruction on how to address the resulting ambiguity. Although we are not aware of conflicts in any of the peer feedback intervention groups in our study, generally, virtual teams appear to be more prone to conflicts than face-to-face groups (Mortensen and Hinds, 2001). Again, this issue may require prior instruction.

As a general remark, we endorse preregistrations for all future studies in this field. This way, researchers’ degree of freedom in the statistical analyses can be limited, thereby increasing the credibility of findings (Simmons et al., 2011; Gelman and Loken, 2013; Chambers and Tzavella, 2022).

## Data Availability Statement

The raw data supporting the conclusions of this article will be made available by the authors, without undue reservation.

## Ethics Statement

Ethical review and approval was not required for the study on human participants in accordance with the local legislation and institutional requirements. The patients/participants provided their written informed consent to participate in this study.

## Author Contributions

HB designed the interventions, conceptualized the study design, and organized the data collection under the supervision of BS. PL and HB performed the statistical analyses. HB wrote the first draft of the manuscript, PL and BS provided the feedback. All authors contributed to manuscript revision, read, and approved the submitted version.

## Conflict of Interest

The authors declare that the research was conducted in the absence of any commercial or financial relationships that could be construed as a potential conflict of interest.

## Publisher’s Note

All claims expressed in this article are solely those of the authors and do not necessarily represent those of their affiliated organizations, or those of the publisher, the editors and the reviewers. Any product that may be evaluated in this article, or claim that may be made by its manufacturer, is not guaranteed or endorsed by the publisher.
